# Geographic variation in the aetiology, epidemiology and microbiology of bronchiectasis

**DOI:** 10.1186/s12890-018-0638-0

**Published:** 2018-05-22

**Authors:** Ravishankar Chandrasekaran, Micheál Mac Aogáin, James D. Chalmers, Stuart J. Elborn, Sanjay H. Chotirmall

**Affiliations:** 1 Lee Kong Chian School of Medicine, Nanyang Technological University, Clinical Sciences Building, 11 Mandalay Road, Singapore, 308232 Singapore; 2 Division of Molecular and Clinical Medicine, School of Medicine, Ninewells Hospital and Medical School, Dundee, UK; 3 Imperial College and Royal Brompton Hospital, London, UK; 4 Queen’s University Belfast, Belfast, UK

**Keywords:** Bronchiectasis, Microbiome, Mycobiome, *Pseudomonas aeruginosa*, Fungi, *Aspergillus spp.*

## Abstract

Bronchiectasis is a disease associated with chronic progressive and irreversible dilatation of the bronchi and is characterised by chronic infection and associated inflammation. The prevalence of bronchiectasis is age-related and there is some geographical variation in incidence, prevalence and clinical features. Most bronchiectasis is reported to be idiopathic however post-infectious aetiologies dominate across Asia especially secondary to tuberculosis. Most focus to date has been on the study of airway bacteria, both as colonisers and causes of exacerbations. Modern molecular technologies including next generation sequencing (NGS) have become invaluable tools to identify microorganisms directly from sputum and which are difficult to culture using traditional agar based methods. These have provided important insight into our understanding of emerging pathogens in the airways of people with bronchiectasis and the geographical differences that occur. The contribution of the lung microbiome, its ethnic variation, and subsequent roles in disease progression and response to therapy across geographic regions warrant further investigation. This review summarises the known geographical differences in the aetiology, epidemiology and microbiology of bronchiectasis. Further, we highlight the opportunities offered by emerging molecular technologies such as -omics to further dissect out important ethnic differences in the prognosis and management of bronchiectasis.

## Background

**Fig. 1:**
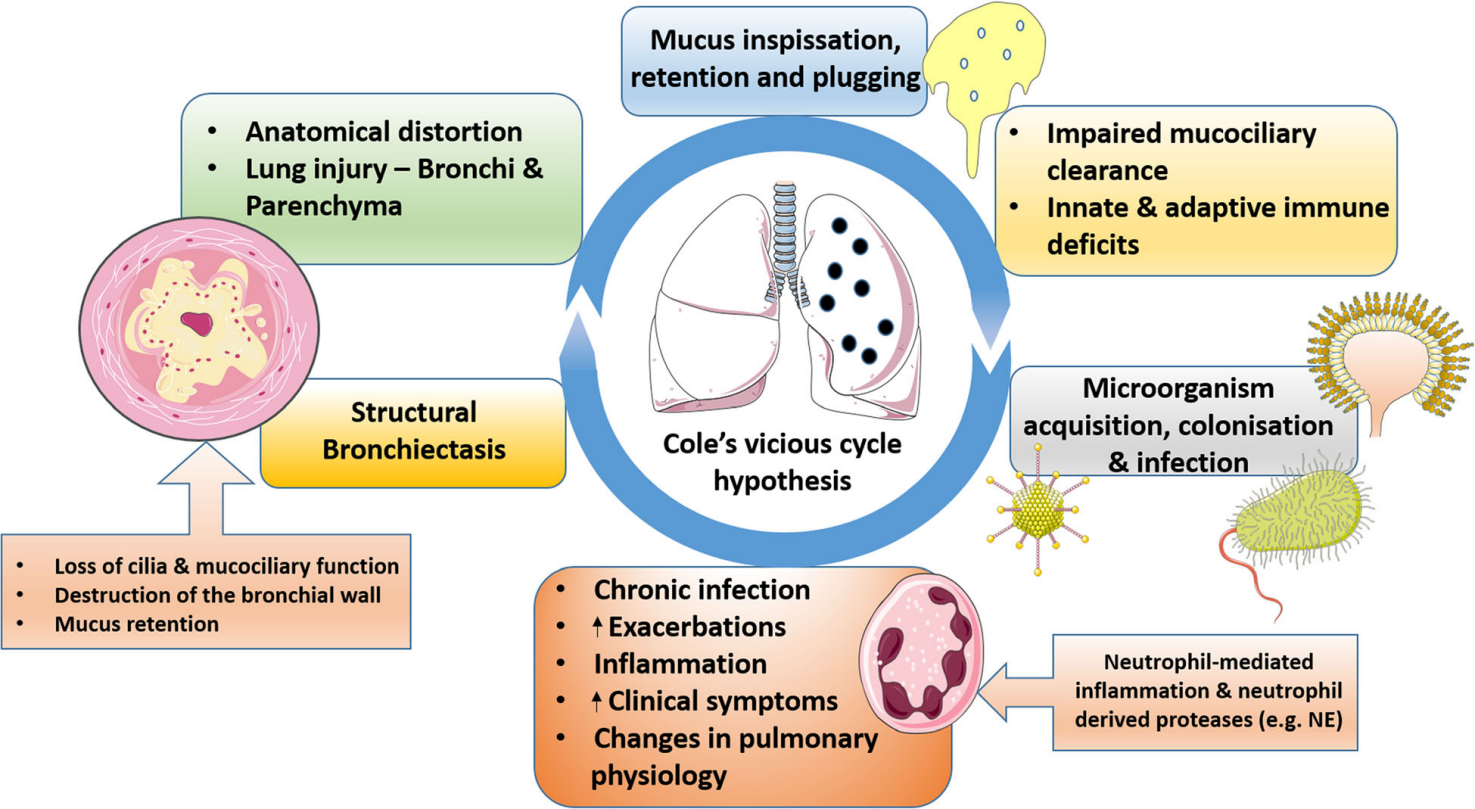
A modern interpretation of Cole’s vicious cycle hypothesis. Abbreviations: NE – Neutrophil elastase, ↑ - Increased

Bronchiectasis is a major chronic pulmonary disease characterised by infection, inflammation and a permanent, irreversible dilatation of the bronchial wall. The interaction of chronic infection, exacerbations and inflammation drive a vicious cycle resulting in lung injury to the bronchi and lung parenchyma. This model proposed by Cole is not well understood in terms of the underlying biology but includes deficits in mucociliary clearance and innate and adaptive immunity (Fig. 1**)**. There is amplification of injury processes following anatomical damage to the bronchi leading to progressive worsening of pulmonary physiology and symptoms with associated increase in exacerbations [1]. The host immune response to infection is primarily neutrophilic and neutrophil derived proteases are deleterious and result in further pulmonary damage amplifying a recurrent cycle [2] (Fig. 1).

### Literature search strategy

A PUBMED review of all articles mentioning the keyword “bronchiectasis” in combination with “epidemiology” or “microbiology” published between 1997 and 2017 was performed. As bronchiectasis in Cystic Fibrosis (CF) represents a separate disease entity in its own right, retrieved articles dealing exclusively with CF-associated bronchiectasis were excluded, as were original articles without radiological confirmation of bronchiectasis. Studies of both adult and paediatric populations were considered and appropriately included.

### Ageing and its impact on bronchiectasis

Bronchiectasis is an age-associated disease [3]. A marked increase in prevalence, particularly of severe disease is observed in the elderly [4]. The global shift in ageing will continue to influence the burden of bronchiectasis, its disease epidemiology and implications for the healthcare systems that provide therapy [5]. In many chronic lung diseases there is an age-related increased prevalence given the multifactorial impact of the aging process on respiratory physiology. Physiological change including decreased diaphragm strength, reduced breathing efficiency and vital capacity (VC) coupled to increases in residual volume (RV) all have important influences on the diagnosis and interpretation of pulmonary function testing (PFTs) across a variety of respiratory pathologies as described by our group and others [6–8]. The diminution of swallowing reflexes and increased prevalence of GORD in the elderly may contribute to the development of bronchiectasis due to subclinical microaspiration including the nasopharyngeal microbiota [9]. Elderly people have more severe disease and atypical presentation with poorer outcomes compared to younger cohorts [10]. Age-associated disease manifestations also correlate closely with variation in immune and microbiome signatures that are associated with the ageing process itself [11, 12]. The immune system, and potentially the microbiome, also undergoes its own change with age, a process incompletely understood, termed ‘immunosenescence’ [13, 14]. Although immunosenescence influences a variety of respiratory disease states, little is known about its effects on bronchiectasis [15]. Nevertheless, associations between lung function decline, infection and age suggest that immunosenecence and potentially bronchiectasis pathogenesis are likely inter-related [16]. Immunosuppression due to leukaemias and their treatment are also interestingly associated with bronchiectasis, a relevant observation for elderly populations [17]. Age-associated pathways including WNT signalling, mTOR and Toll-like receptors (TLRs) all have possible roles in COPD and IPF pathogenesis and could explain age-associated severity in bronchiectasis. Telomere dysfunction and senescence associated pathways have been described in explants studied from patients with bronchiectasis [18]. As such, this represents an important area of future interest and research [19–21].

### Geographic variation in the aetiology of bronchiectasis

#### Bronchiectasis in children vs adults

A increased risk of non-CF bronchiectasis is observed at the extremes of age with children under 5 years and adults over 75 years of age at greatest risk of disease [22]. Particular aetiologies and clinical manifestations are observed in childhood bronchiectasis, which more frequently includes primary and secondary immunodeficiency, ciliary dyskinesia, congenital malformations, bronchiolitis obliterans and skeletal disease [23]. As with adult bronchiectasis, infection is highly associated with disease and those with childhood bronchiectasis are at increased risks of more severe disease in later life [24]. While the most striking incidence of childhood bronchiectasis is seen in indigenous populations including Maori and Pacific Islanders of New Zealand, Australian aboriginal and Alaskan native children, increasing rates have also been observed outside these at risk populations [25]. It is difficult from the current literature to discern if the broader global shifts in bronchiectasis prevalence are due to ‘true’ changes in our understanding of aetiology, including that in childhood or alternatively a better awareness of the disease, a development of more recent times.

### Bronchiectasis in Europe

Cystic fibrosis (CF), caused by dysfunction or absence of the Cystic Fibrosis Transmembrane Conductor Regulator protein (CFTR) genetically predisposes those affected to bronchiectasis; but this condition is most prevalent in Caucasian populations and is less commonly encountered in Asians. In Europe, North America, Australian and New Zealand, neonatal screening is widely available and most people with CF are diagnosed soon after birth. The majority of non-CF bronchiectasis in studies reported from Europe, Australia and the USA have no identifiable aetiology and is labelled idiopathic [3, 26]. As infection is crucial in the pathophysiology of bronchiectasis, it is unsurprising that post-infection bronchiectasis is the most commonly identifiable cause for disease development. Infection with *Mycobacterium tuberculosis*, non-tuberculosis mycobacteria (NTM), childhood *Bordetella pertussis* (whooping cough) and viruses including influenza, measles and adenovirus, have all been implicated in post-infection bronchiectasis states. It is however, in many such cases, difficult to be certain of this aetiology because of recall bias from events often many decades in the past. Importantly, COPD, asthma, connective tissue disease and immunodeficiency are all noted as important potential contributing factors among European patients [3, 27]. Gender seems to additionally exert an effect on particular aetiologies with males more likely to exhibit COPD and females more likely to exhibit asthma-related aetiologies [3]. European patients with COPD also tended to be older while immunodeficiency, ciliary dysfunction and irritable bowel disease (IBD) were all observed in younger patients [3]. The co-morbidities seen most commonly in Europe include COPD, asthma and IBD; all representing independent mortality risk factors in those with non-CF bronchiectasis [27]. COPD-associated bronchiectasis is a leading cause in Europe [3, 28–30] with allergic reactions to fungi belonging to the genus *Aspergillus* (Allergic bronchopulmonary aspergillosis - ABPA) particularly notable in United Kingdom (UK) based cohorts [28, 31, 32].

### Bronchiectasis in the Americas

Bronchiectasis caused by immune-related mechanisms including autoimmunity, immunodeficiencies and hematologic malignancies were identified as predominant aetiologies in the United States [33]. This work demonstrates a low rate of idiopathic bronchiectasis and importantly reveals that systematic evaluation may identify an aetiology in a high proportion of cases suggested by an earlier UK study [31]. In the US, immune dysfunction was frequently associated with bronchiectasis including that among stem-cell transplant recipients who suffered graft versus host disease [33]. Outside of indigenous Canadian cohorts, where high rates of childhood bronchiectasis are reported, data on aetiology of adult Canadian non-CF bronchiectasis is rather limited and the precise nature of aetiology in this country is largely uncertain [34, 35]. In Latin America aetiology is, like elsewhere, driven by infection and influenced by infectious disease epidemiology such as that in endemic TB regions or against backdrops of higher rates of pertussis and measles which in turn relate to the lower vaccine uptake rates. Higher rates of pneumonia and tuberculosis in childhood are also likely key contributing factors to bronchiectasis in this region [36].

### Bronchiectasis in the Asia-Pacific region

The true prevalence of bronchiectasis in communities in the Asia-Pacific region is largely unknown and should be considered a potential diagnosis in all populations. Important aetiologies of bronchiectasis seen in other regions including immunodeficiency syndromes such as, common variable immunodeficiency, secondary immunoglobulin disorders (frequently drug related) and mucociliary defects including primary ciliary dyskinesia, chronic aspiration, autoimmune/connective tissue diseases, particularly rheumatoid arthritis, and ABPA are described and in some cases result in a delayed diagnoses. In Japan, a less studied inflammatory disease, sinobronchial syndrome is documented in many cases of bronchiectasis [37].

**Fig. 2:**
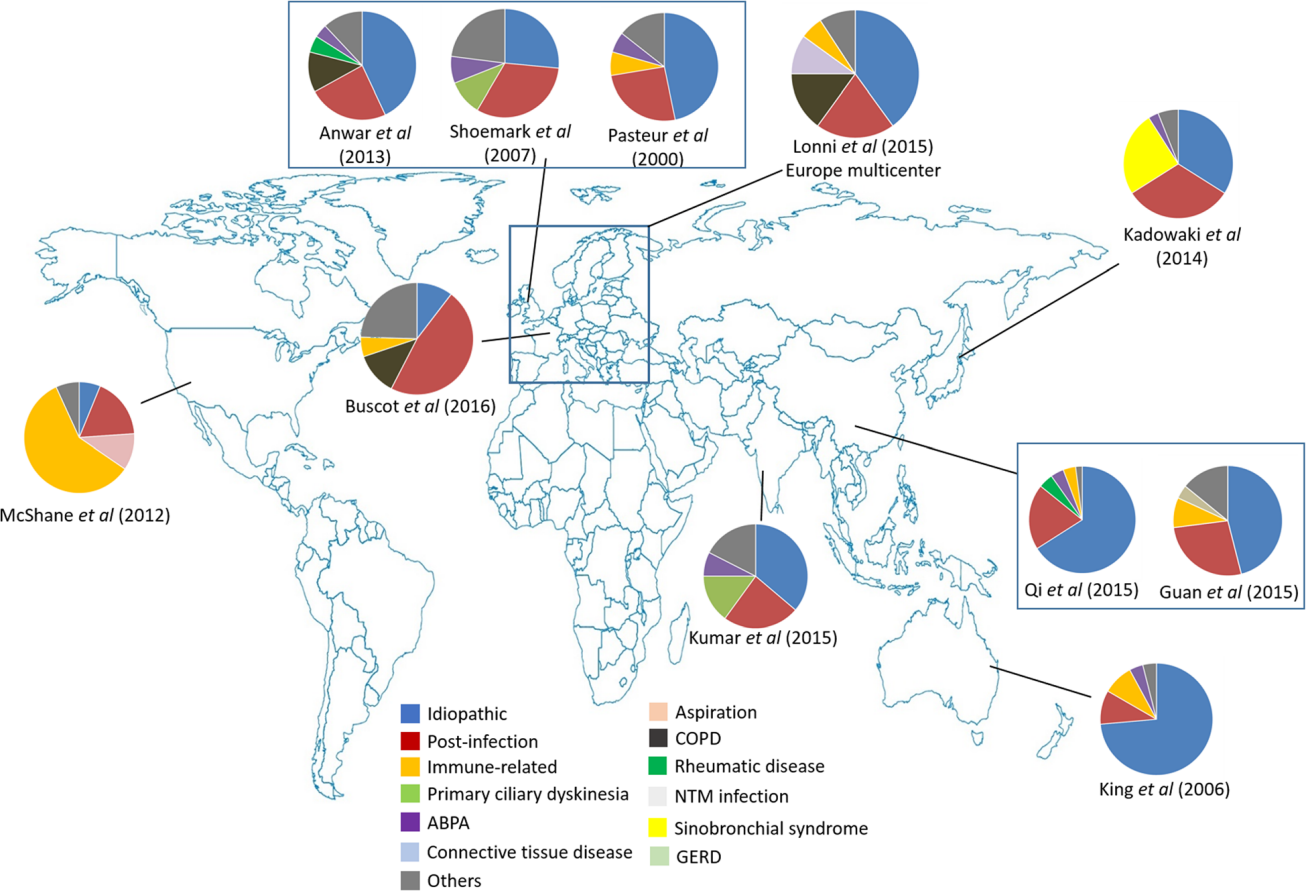
Predominant aetiologies across different geographic regions and ethnic populations. The individual pie charts indicate the top aetiologies (top 4 or 5) in each cohort. Abbreviations: ABPA – Allergic Broncho-Pulmonary Aspergillosis, COPD – Chronic Obstructive Pulmonary Disorder, NTM – Non-Tuberculosis Mycobacteria, GERD – Gastro-Esophageal Reflux Disease

While geographic variation in bronchiectasis aetiology is described, selection or referral biases, and, the extent of testing to seek a diagnosis of bronchiectasis in individual patients may have resulted in the observed patterns in the populations reported. Figure 2 illustrates the existing literature of available studies focused on bronchiectasis aetiology based on geography.

### Geographic variation in the epidemiology of bronchiectasis

#### Bronchiectasis in children vs adults

The most striking variation in bronchiectasis epidemiology is observed among indigenous children of Australia, Alaska, Canada and New Zealand [34, 35, 38–40]. Here, paediatric populations exhibit exceptionally high rates compared to non-indigenous groups with infant or childhood pneumonia cited as the primary cause in many cases. These combined observations point to the contribution of genetic predisposition, early childhood infection and overall lower socio-economic status as important features in pathogenesis particularly among specific indigenous populations [25]. Considering the Pacific region; a high incidence is observed in children under 15 years of age in New Zealand and substantial differences noted within their indigenous ethnic groups and across their geographic regions [41]. Most paediatric bronchiectasis in New Zealand is idiopathic with predominant chronic *Haemophilus influenzae* infection which in turn associates with reduced lung function [42]. Bronchiectasis in children is also associated with high rates of hospital admission particularly in Australian aboriginal children. This latter group have one of the highest reported prevalence rates of bronchiectasis (14.7 per 1000) worldwide [43, 44]. In separate work, Alaskan native children are described to have extremely high rates of bronchiectasis compared to other populations and, in most of these individuals, infant or childhood pneumonia is the primary cause of disease [38–40]. All the aforementioned patient groups are clearly enriched by disease occurrence, an important feature that offers the opportunity for research to better understand the roles and interaction of genetic predisposition and early childhood infection to the subsequent development of bronchiectasis.

#### Bronchiectasis in Europe

Incidence and prevalence rates of bronchiectasis in the UK have increased annually from 2004 and are associated with significant mortality [4]. Studies from the UK’s North East (*n* = 189) illustrate that occurrence of idiopathic bronchiectasis is high and that those identified with post-infective aetiology developed the condition earlier in life [28]. In contrast, a Greek study (*n* = 277) demonstrated that prior tuberculosis, pertussis, measles and pneumonia were the leading causes of bronchiectasis [45]. A retrospective study from Nice in southern France (*n* = 311) similarly described high rates of post-infectious (mainly post-tuberculous) bronchiectasis [29]. Despite these country-based reports, a large multicentre dataset (*n* = 1258) collated from across Europe (Monza, Italy; Dundee and Newcastle, UK; Leuven, Belgium; Barcelona, Spain; Athens, Greece and Galway, Ireland) illustrated that most patients have idiopathic disease. Among identifiable causes of bronchiectasis, post-infectious did however remain the commonest. Interestingly in this large dataset, COPD-related bronchiectasis was associated with a higher Bronchiectasis severity index (BSI) [3].

In Germany (2005–2011) the prevalence of bronchiectasis was 67 cases per 100,000; associated with concomitant increases in hospital admissions and an increased incidence with age [46, 47]. A large population based study in Catalonia (north-eastern Spain) similarly found high prevalence (36.2 cases per 10,000) and incidence rates (4.81 cases per 10,000). In contrast to other global datasets, the prevalence and incidence of bronchiectasis in this study was highest in older males [48]. A larger multicentre study in Spain however showed contrasting results with higher prevalence in females and elevated rates of post-infectious disease [30]. Of interest, greater hospital admissions and treatment costs per patient in Spain were inversely related to bronchiectasis where it was the primary diagnosis but increased when identified as a secondary diagnosis clearly highlighting a need for focus on earlier diagnosis [49]. Northern European countries such as Finland interestingly report a lower incidence of bronchiectasis compared to worldwide estimates. This is also accompanied by lower hospitalisation and mortality rates from the disease [50, 51]. Overall, these data clearly illustrate the changing and variation in epidemiology and aetiology of bronchiectasis even within Europe which in turn contrasts to that in the Americas and Asian sub-continents.

#### Bronchiectasis in the Americas

Seitz et al. (2012) reported an annual increase of 8.7% in the prevalence of bronchiectasis in the US with a higher prevalence in Asian Americans when compared to European and African Americans. This was based on thoracic computed tomography (CT) scans [52]. Similar increases in bronchiectasis incidence was described between 2009 and 2013 with high rates in women and the elderly [53]. McShane et al. (2012) further illustrated that ethnicity was one of the major contributing factors for the observed aetiological differences in disease, an important consideration for clinicians in an increasingly multi-ethnic resident population across different countries. Rheumatoid Arthritis (RA) was interestingly a common aetiology in African Americans and hematologic malignancies more common in European Americans in this study. Subsequent work also supports the association between hematologic malignancy and bronchiectasis while the role of connective tissue disorders is also corroborated by several investigations [17, 54]. The first report from the US bronchiectasis research registry was recently published and characterised 1826 patients. Its results concurred with others and illustrated a higher occurrence in women. Within the analysed cohort, a higher prevalence of the disease was described in European Americans [55]. The status of bronchiectasis as a largely under-studied disease is further reflected by the relative lack of prevalence data from Canada, the Caribbean and South America, where further studies are warranted.

#### Bronchiectasis in the Asia-Pacific region

In the Asian subcontinent, considerable gaps in our understanding of bronchiectasis epidemiology continue to exist. No comprehensive prevalence datasets for either China or India are currently available however work is currently ongoing to address this. There are sporadic regional reports available that provide some insight into bronchiectasis in this highly affected region.

A recent pan-Indian study (*n* = 680) identified post-infection (41%) to be the primary cause for bronchiectasis with post-tuberculous disease identified as the predominant aetiology (29.8%), whilst ABPA is the most common cause after this and identified in 12% of Indian cases [56]. An aetiological study across different ethnicities in the Guangzhou region of mainland China (*n* = 148) identified idiopathic bronchiectasis (45%) as the most common cause with the high rates of disease related to post-infection (27%) also noted [57]. Among the Han population of mainland China (*n* = 476), rates of idiopathic bronchiectasis (66%) are even more striking and followed by post-tuberculosis as the most prevalent aetiologies observed (16%) [58].These Chinese studies illustrate that whilst post-tuberculous bronchiectasis remains important in Asia, idiopathic bronchiectasis is also highly prevalent. In a small study from Hong Kong (*n* = 100), idiopathic disease dominates (82%) and patients with bronchiectasis are mainly female with high hospitalisation and mortality rates; 21.9 cases per 100,000 and 2.7 cases per 100,000 respectively [59, 60].

In contrast to China however, work from Thailand (*n* = 50) indicates that post-infection related bronchiectasis and specifically post-tuberculosis associated disease was commonest. Similarly, a high prevalence of post-infectious bronchiectasis was reported in Indian children (*n* = 80) followed by primary ciliary dyskinesia and ABPA [61, 62]. A high prevalence of bronchiectasis is reported in South Korea (*n* = 1409) and in one particular study of respiratory patients, 9% were deemed to have bronchiectasis with higher prevalence in females [63].

A variety of reasons may be put forward to explain the outlined epidemiological differences in bronchiectasis that exist across Europe, the Americas and the Asia-Pacific. For example, tuberculosis is rare in more developed countries when compared to the Asia-Pacific or Africa potentially explaining the high frequencies of post-tuberculous disease found in these regions. Potential genetic predisposition to bronchiectasis may account for the increased disease prevalence in indigenous communities in the Asia-Pacific region. The influence of the environment and its accompanying climate may also influence microorganisms and/or pathogens that affect the bronchiectasis airway. Hence, we next outline geographic variations in the airway microbiology in bronchiectasis which in itself may account for some of the observed differences in epidemiological patterns of disease.

### Geographic variation in the microbiology of bronchiectasis

#### The Bacteriome

*Pseudomonas aeruginosa* and *H. influenzae* are the most common bacteria detected in bronchiectasis airways globally although proportions vary among the different populations [45, 64]. Other bacterial genera described in bronchiectasis airways include *Streptococcus*, *Prevotella*, *Veillonella* and *Staphylococcus* [65–67]. *P. aeruginosa* is associated with poorer pulmonary function, higher hospitalisation rates and greater morbidity and mortality compared to *H. influenzae* [68–78].

Non-tuberculosis mycobacteria (NTM) are another important group of organisms that frequently infect the airway in adult bronchiectasis. Bronchiectasis and NTM are highly associated pulmonary diseases with airway distortion predisposing to NTM infection [79, 80]. While NTM is isolated from the bronchiectasis airway and clearly associates with poorer outcomes and more aggressive disease in most cases (largely dependent on the species involved), in some studies, it interestingly has been associated with a milder phenotype, less severe disease, lower exacerbations and better pulmonary function [81, 82]. NTM colonisation in common with *P. aeruginosa* is more frequent in older patients with gender preponderance for postmenopausal women and a lower prevalence is observed in paediatric populations [82–85]. *Mycobacterium avium complex* (MAC) is generally the most common form affecting bronchiectasis patients although geographic variation exists [80, 82, 84, 86].

#### The bronchiectasis bacteriome in children vs adults

Studies in children focused on bronchiectasis microbiology highlight *H. influenzae* as the most prevalent sputum organism (30–83%) from work originating in New Zealand. Of note, *P. aeruginosa* largely considered an airway organism affecting adults was described in up to 4% of children with bronchiectasis with *S. pneumoniae* (5–14%) and *M. catarrhalis* (2–8%) also described [41, 42, 85]. Several studies, some using bronchoalveolar lavage (BAL) from indigenous children in Northern Australia, showed marked similarity for their microbiology compared to the New Zealand datasets except that none of the children in this latter work were *P. aeruginosa* positive [87, 88]. When compared to European paediatric data from the UK and Ireland; children were found to have similar dichotomy between *H. influenzae* and *P. aeruginosa* in the airway and also high detection of *S. pneumoniae* [89–91]. There are however some notable intra-country differences in geographic patterns for *P. aeruginosa*: low levels in Newcastle compared to higher levels in London (5% versus 11% respectively) which contrasts to *M. catarrhalis* where occurrence in Newcastle is higher than that in London [90, 91]. Such differences may reflect differing referral patterns or presence of specialist clinics at particular centres but nonetheless serve to highlight the spectrum of disease heterogeneity seen in children. When evaluated against data from an adult bronchiectasis population in the UK, expectedly higher rates of *P. aeruginosa* (49%) are observed compared to the paediatric cohorts [70]. Taken together, these observations suggest that variation in paediatric bronchiectasis microbiology may be more complex than that in adults and illustrate within-country differences in addition to geographic and continental variation.

#### The bronchiectasis bacteriome in Europe

In European studies of the bacteriome in adult bronchiectasis, data combining Spanish and Scottish datasets illustrate equal proportions of *H. influenzae* and *P. aeruginosa* with *E.coli* interestingly isolated from a tenth of the studied cohort [92]. Separate work from Greece, Belgium and France concur with other European studies detecting high rates of airway *P. aeruginosa* and *H. influenzae* but low NTM [29, 45, 47, 93]*.* An important study, using 16 s rRNA sequencing from Northern Ireland showed that change to bacterial communities in the bronchiectasis airways may not in fact be a driver for exacerbations however a trend toward lower microbial diversity was described. In terms of relative abundance, *Haemophilus spp.* dominates *Pseudomonas spp.* in stable patients and post-antibiotic treatment, a mild increase in anaerobic bacteria is seen with a corresponding decrease in aerobes [94]. In contrast however, other 16 s rRNA datasets assessing both the stable and exacerbation states found that *P. aeruginosa* was the commonest organism in both categories [65]. More recent studies, also from the UK, have reaffirmed the important original observations that changes from a stable to exacerbation state involves more than a simple alteration in the bronchiectasis airway bacteriome [67]. While it may be too early to speculate on specific patterns of microbes and an association to exacerbations, data in support of this hypothesis is the observation (from pyrosequencing UK datasets) that an inverse relationship does exist between airway abundance of *P. aeruginosa* and *H. influenzae* in the bronchiectasis airway and that specific microbial patterns do associate with the exacerbation state [71]. Sequencing approaches have also interestingly shown that long-term erythromycin treatment adversely affects *H. influenzae-*dominant patients by increasing the relative abundance of *P. aeruginosa* [66, 95].

#### The bronchiectasis bacteriome in the Americas

Varying rates of colonisation by *P. aeruginosa* are described across varied ethnic groups in the US with Hispanic Americans having the highest rates, followed by European Americans and African Americans [33]. In more recent data from the US Bronchiectasis research registry (*n* = 1826) however, NTM were found to be most frequent (54%) with MAC followed by *M. abscessus* and *M. chelonae* being the commonest isolated NTM species. *P. aeruginosa* was described in one-third and *S. aureus* in one-eighth of patients with colonisation by either of these species less in patients affected by NTM. Patients with detectable NTM also developed bronchiectasis later and were predominantly female [55]. Studies from Europe have found similar discordance between NTM and these other bacteria in the bronchiectasis airways [96]. Of note, however, is the ascertainment bias in the US bronchiectasis research registry: many are tertiary referral centres with NTM referral patterns potentially skewing the reported data. It is likely that the US does however have more NTM-associated disease overall in comparison to other geographic regions however the current available datasets don’t permit us to definitively establish this.

#### The bronchiectasis bacteriome in the Asia-pacific region

In Asia, similar patterns, in both the ethnic Han population from Shandong province (eastern China) and the southern Chinese city of Guangzhou are observed with predominance of *P. aeruginosa* and *H. influenzae* with colonisation rates of the former stable across the different bronchiectasis aetiologies identified in these populations. NTM rates unlike the US were low in Chinese studies [57, 58]. Prospective work from Thailand found similar patterns to that described in China although in this population *Klebsiella pneumoniae* was detected in equal proportions to *H. influenzae* [61]. South Korea has a different distribution with high NTM (44.5%), similar to that of the US and lower rates of *P. aeruginosa* (18.1%). Like the Thai patients, South Koreans also had a significant prevalence of *K. pneumoniae* [97]. While geographically close; work from Japan however reports *P. aeruginosa* as the predominant airway bacteria (24%) closely followed by only moderate levels of NTM (19%) [37]. Interestingly, in the Pacific region, specifically central and southern Australia; reported rates of *H. influenzae* (36–81%) compared to *P. aeruginosa* (7–26%) are higher with very low occurrences of NTM (1–2%) [88, 98, 99].

A higher mean relative abundance of *Haemophilus spp.* compared to *Pseudomonas spp.* was reported in an Australian study. The authors propose a bacteriome based patient stratification system to predict exacerbations in bronchiectasis. In this system, patients with an airway bacteriome dominated by *P. aeruginosa* or *Veillonella spp.* experience higher rates of future exacerbations compared to patients whose airways are dominated by *H. influenzae* [100]. In addition, *H. influenzae* dominant individuals experience milder disease in contrast to *P. aeruginosa* which may be attributed to competitive exclusion between the organisms [101]. While interesting, these observations are importantly derived from datasets from the BLESS trial that assessed patients with a history of at least two exacerbations per year. Therefore, these identified patterns were based on comparisons between ‘very frequent’ to ‘less frequent’ exacerbators and lacked assessment against non-exacerbators.

While culture based detection of airway bacteria is routinely used in bronchiectasis, next-generation sequencing (NGS) approaches are being used in research as a faster and more robust alternative for identifying airway pathogens [65, 66, 102]. Such culture-independent sequencing methodologies have been applied in bronchiectasis and identify a greater degree of airway microbial diversity (Table 1**)** [103, 104]. These methods are not yet appropriate for clinical use because of the challenges in bioinformatic analysis and standardisation. This will be efficiently computerised in the coming years and facilitate clinical translation. In spite of the increasing exploration of the bacteriome using such technological advances, exploration of viral and fungal residents of the lung and their association with bronchiectasis has lagged behind. While the small number of available studies limits our understanding of viral and fungal contributions to bronchiectasis and their geographic variability, we nonetheless review below their currently understood respective contributions and the evidence supporting their clinical association with bronchiectasis.

**Table 1:** Predominant pathogens identified in bronchiectasis cohort studies

Method			Population	Sample size	Predominant pathogens (by sequencing)	Predominant pathogens (by culture)	Ref
Sputum culture	BAL culture	16S rRNA sequencing					
✓			Adult	*n* = 123	N.A.	*P. aeruginosa* *H. influenzae* *M. avium intracellulare* *S. pneumoniae* *S. aureus*	[128]
✓			Adult	*n* = 100	N.A.	*P. aeruginosa* *H. influenzae* *S. pneumoniae* *S. aureus* *M. catarrhalis*	[74]
✓			Adult	*n* = 193	N.A.	*H. influenzae* *P. aeruginosa* *M. catarrhalis* *S. pneumoniae* *S. aureus* *A. fumigatus*	[32]
✓			Adult	*n* = 155	N.A.	*H. influenzae* *P. aeruginosa* *S. pneumoniae* *M. catarrhalis* *S. aureus*	[70]
✓	✓		Adult	*n* = 77	N.A.	*H. influenzae* *S. pneumoniae* *P. aeruginosa*	[129]
	✓		Children	*n* = 113	N.A	*NTHi* *S. pneumoniae* *M. catarrhalis* *S. aureus* *P. aeruginosa*	[88]
✓			Adult	*n* = 89	N.A.	*H. influenzae* *P. aeruginosa* *M. catarrhalis* *S. pneumoniae* *S. aureus* *Aspergillus* spp. *M.avium* complex	[69]
		✓	Adult	*n* = 11	*P. aeruginosa* *Prevotella spp.* *Streptococcus spp.* *Haemophilus spp*	N.A.	[65]
		✓	Adult	*n* = 41	*H. influenzae* *P. aeruginosa* *S. pneumoniae* *S. aureus* *M. catarrhalis*	N.A.	[66]
✓		✓	Adult	*n* = 70	*Pseudomonadaceae* *Pasteurellaceae* *Streptococcaceae*	*P. aeruginosa* *H. influenzae*	[71]
✓		✓	Adult	Culture: Stable: *n* = 40 Exacerbation : *n* = 11 Sequencing: Stable: *n* = 10 Exacerbation : *n* = 19	*Haemophilus spp.* *Pseudomonas* spp. *Streptococcus spp.* *Achromobacter spp*	**Stable patients:** *P. aeruginosa* *H. influenzae* *Prevotella spp.* *Veillonella spp.* **Exacerbation patients:** *P. aeruginosa* *H. influenzae* *S. pneumoniae* *Methicillin-resistant* *S. aureus*	[94]
✓		✓	Adult	Stable *n* = 76, *n* = 64/76 patients followed-up during exacerbation.	*Hemophilus spp.* *Pseudomonas spp.* *Streptococcus spp.*	*P. aeruginosa* *S. aureus* *H. influenzae*	[67]

The list order of pathogens corresponds to frequency of identification. Abbreviations: *P. aeruginosa* – *Pseudomonas aeruginosa*, NTM – Non-Tuberculosis Mycobacteria, *H.influenzae* – *Haemophilus influenzae*, NTHi – Non-typeable *Haemophilus influenzae*, *C. albicans* – *Candida albicans*, *S. pneumoniae* – *Streptococcus pneumoniae, S. aureus* – *Staphylococcus aureus*, *M. catarrhalis* – *Moraxella catarrhalis*, *A. fumigatus* – *Aspergillus fumigatus*, *M. avium* – *Mycobacterium avium*

### The Virome

**Fig. 3:**
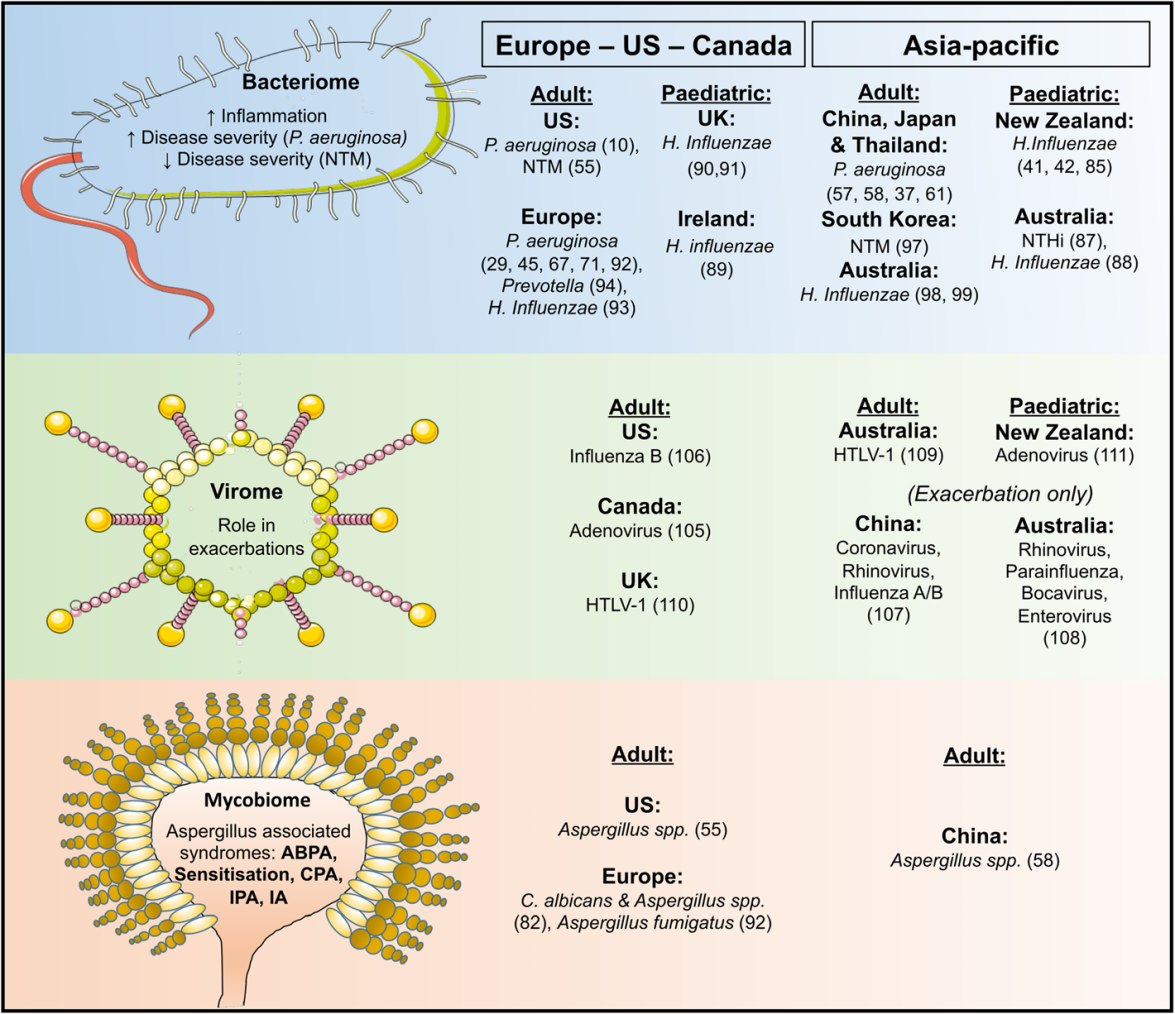
Differences in the microbiome between Europe, the US and the Asia-Pacific by sputum culture illustrating the predominant organisms in stable states and viruses only during exacerbations. The bacteriome contributes to host inflammation and disease severity, the virome in exacerbations and the mycobiome is an understudied group with potential clinical impact. Abbreviations: US – United States, UK – United Kingdom, *P. aeruginosa* – *Pseudomonas aeruginosa*, NTM – Non-Tuberculosis Mycobacteria, *H.influenzae* – *Haemophilus influenzae*, NTHi – Non-typeable *Haemophilus influenzae*, HTLV-1 – Human T-Lymphotropic Virus type 1, *C. albicans* – *Candida albicans*, ABPA – Allergic Broncho-Pulmonary Aspergillosis, CPA – Chronic Pulmonary Aspergillosis, IPA – Invasive Pulmonary Aspergillosis, IA – Invasive Aspergillosis ↑ - Increased, ↓ - Decreased

Our current understanding of the virome in bronchiectasis is limited and most studies of viruses in bronchiectasis are rarely assessed compared to the baseline presence of viruses in healthy individuals. Recent work however has suggested a role for viruses in exacerbations of bronchiectasis where bacterial density and diversity remains stable during exacerbations [94]. Early work from the US and Canada were the first to report viral infection, specifically Influenza B and adenovirus in bronchiectasis, respectively [105, 106]. More recently, work from China (Guangzhou) reports coronavirus, rhinovirus and influenza A and B detection during exacerbations which is associated with concomitant increases in both airway and systemic inflammation (IL-1β; IL-6) [107]. Systemic and airway TNF-α was also elevated in virus positive exacerbations [107]. Interesting work from Australian indigenous children similarly illustrates an increased viral detection, particularly rhinoviruses during exacerbations. Children positive for virus during an exacerbation are also more likely to be hospitalised [108]. These data however do not elucidate whether viruses are a cause or consequence of exacerbations, an area for future investigation. Despite this, recent work from both Europe and the Asia-Pacific has indicated a potential role for human T-lymphotropic virus type 1 (HTLV-1) mediated inflammation in the causation of bronchiectasis [109, 110]. A separate New Zealand based study similarly proposed adenovirus infection as a potential cause of post-infectious bronchiectasis (Fig. 3) [111].

### The Mycobiome

Our knowledge of the pulmonary mycobiome is less well characterised and although technically challenging, may provide new insight into its potential role in bronchiectasis. Fungi, a separate kingdom of organisms with more than 1.5 million estimated species requires dedicated study in bronchiectasis where anatomical distortion to the airways predisposes patients to both acquisition and colonisation by fungi [103, 112–115]. Those belonging to the Ascomycota phyla (e.g. Aspergillus *spp.*) form spores and through inhalation, on a daily basis, thousands of fungal spores have access to the airways [103]. Dependant on the underlying state of host immunity, disease can result and, manifestations range from allergic (in immune hyper-reactivity) to invasive (in severe immunodeficiency). Such disease variation is best characterised by *Aspergillus-*associated syndromes outlined in Fig. 3. Allergic bronchopulmonary aspergillosis (ABPA) is a recognised aetiological factor for the occurrence of bronchiectasis while sensitisation increases the incidence of bronchiectasis in asthmatics [116–119].

In addition to *Aspergillus*, *Candida spp.* represents another fungal genus of potential importance, one routinely cultured from airway samples. Importantly, *Candida spp*. are abundant in the oral cavity even of healthy individuals and hence whether they represent genuine respiratory colonisers and/or pathogens in bronchiectasis remains uncertain [120].

A great paucity of data exist specifically assessing fungi in the airways of patients with bronchiectasis. Most studies of bronchiectasis don’t specifically include dedicated fungal culture and most published reports are based on their incidental detection. As documented by recently published ‘research priorities in bronchiectasis’ from the EMBARC collaboration, work addressing fungi is both necessary and of importance in bronchiectasis [121]. A Spanish study reports that *Aspergillus* and *Candida spp.* together contribute the highest proportion of fungi isolated by culture from the bronchiectasis airway. Within the *Aspergillus* genus, *A. fumigatus* is the most common coloniser and other filamentous fungi such as *Penicillium*, *Scedosporium* and *Fusarium* are less frequently seen. Critically, chronic antibiotic use in this work was associated with prolonged colonisation by these fungi [82]. Data from the US bronchiectasis research registry (*n* = 1826) reports an incidence of 19% of *Aspergillus spp.* in their population [55] Two separate studies from the UK illustrate that *A. fumigatus* colonisation and/or sensitisation is positively correlated with NTM occurrence. The co-existence of chronic pulmonary aspergillosis and NTM infection predicts mortality in bronchiectasis [122, 123]. Culture-based identification, part of the routine diagnostic microbiology work up in bronchiectasis is inefficient for fungal detection because most fungal species do not grow on common laboratory media [124]. To overcome this, work employing next-generation sequencing (NGS) such as targeted amplicon sequencing and whole-genome shotgun metagenomics may reveal the true diversity of fungal microorganisms within the microbiome that may colonise and contribute to pulmonary pathology in bronchiectasis and as such should be a focus for future work [103, 104, 125]. Figure 3 summarises the ‘microbiome’ in bronchiectasis that consists of the ‘bacteriome’, ‘virome’ and ‘mycobiome’ where based on country, the predominant organism has been identified and geographical differences outlined between Europe, the US and the Asia-Pacific. Findings relating to adult and paediatric populations are also indicated.

### Geographic variation in clinical bronchiectasis phenotypes

Studies assessing clinical phenotypes in bronchiectasis are lacking. The most extensive study to date included 1145 patients across five databases in Europe and identified four distinct phenotypes: severe *Pseudomonas* infection (16%), other chronic infections (24%), daily sputum production without colonisation (33%) and dry bronchiectasis (27%) [126]. This contrasted with a single reported Asian analysis from China where 148 patients were assessed [127]. Again, four different groups were identified but the only commonality was a severe group with post-infective bronchiectasis and the presence of airway *Pseudomonas*. Other key groups from the Chinese study included mild idiopathic disease in young patients, severe idiopathic disease of late-onset and moderate disease in the elderly. A third study focused solely on the Spanish national database of 468 patients again identified the presence of airway *Pseudomonas* as a separate clinical phenotype [30]. In this setting, it was characterised by severe disease, chronic infection, airflow obstruction and severe exacerbations in elderly men. Geographic variation in bronchiectasis phenotypes is likely very relevant for our understanding of disease pathogenesis according to region and requires further and more detailed study. Importantly, while results from the various cluster studies in bronchiectasis may represent true geographic variation in disease, they are limited by the quality and quantity of data put into the clustering process itself and, has largely remained uncontrolled for referral bias. An overwhelming message across all three studies is that clinical data alone was poor at identifying meaningful patient ‘clusters’ providing a strong argument for alternative approaches including use of “omics” for patient stratification. Perhaps targeted therapeutic approaches in the future, applicable to specific regions and populations may become relevant as we start to decipher the drivers of varying endotypes of disease.

## Conclusion

As the incidence and prevalence rates of bronchiectasis continue to increase with global ageing, it can no longer be considered an ‘orphan’ respiratory disease. Despite its documented economic burden, effects on quality of life, and social implications, bronchiectasis is a relatively neglected pulmonary disease. Further investment and research are now required, that which focuses on ethnic variations and accounts for geographical differences to permit a more ‘personalised’ approach to its diagnosis, management and understanding of prognosis across countries. The recommendations for research priorities in bronchiectasis by the European Multicentre Bronchiectasis Audit and Research Collaboration (EMBARC) stresses the importance of large cohort studies to better understand the varying aetiologies that drive the disease across different populations. Elucidating differences in less studied organisms including fungi and viruses are also highlighted and research focus in these key areas would improve our understanding of disease while permitting a more personalised therapeutic approach perhaps varied by geographic region [121].

Differences in the aetiology, epidemiology and microbiology of bronchiectasis can be observed across countries and continents and may influence the observed clinical phenotypes, which in turn likely influences treatment and outcomes. Studies targeting geographic regions where a paucity of data exists including Asia, Africa and South America are now necessary. If effective treatment approaches are to be realised in bronchiectasis – a condition for which no licenced therapies currently exist – success will likely depend on more targeted approaches that acknowledge the marked geographic variability associated with this heterogeneous disease.

## Abbreviations

ABPA: Allergic Broncho-Pulmonary Aspergillosis
*B. pertussis*: *Bordetella pertussis*
BAL: Broncho-Alveolar Lavage
BLESS: Bronchiectasis and Low-Dose Erythromycin Study
BSI: Bronchiectasis Severity Index
CF: Cystic Fibrosis
CFTR: Cystic Fibrosis Transmembrane Conductor Regulator protein
COPD: Chronic Obstructive Pulmonary Disease
CT: Computed Tomography
*E.coli*: *Escherichia coli;*
EMBARC: European Multicentre Bronchiectasis Audit and Research Collaboration
*H. influenza*: *Haemophilus influenza*
HTLV-1: Human T-Lymphotropic Virus type 1
IL: Interleukin
IPF: Idiopathic Pulmonary Fibrosis
*K. pneumoniae*: *Klebsiella pneumoniae*
*M. abscessus*: *Mycobacterium abscessus*
*M. catarrhalis*: *Moraxella catarrhalis*
*M. chelonae*: *Mycobacterium chelonae*
*M. tuberculosis*: *Mycobacterium tuberculosis*
MAC: *Mycobacterium avium complex*
mTOR: Mechanistic Target Of Rapamycin
NGS: Next Generation Sequencing
NTM: Non-Tuberculosis mycobacteria
*P. aeruginosa*: *Pseudomonas aeruginosa*
PFTs: Pulmonary Function Testing
RA: Rheumatoid Arthritis
RNA: Ribo-Nucleic Acid
rRNA: Ribosomal RNA
RV: Residual Volume
*S. aureus*: *Staphylococcus aureus*
*S. pneumoniae*: *Streptococcus pneumoniae*
spp.: Species
TLR: Toll-Like Receptors
UK: United Kingdom
US: United States
USA: United States of America
VC: Vital Capacity
